# Supplementary material for: Photocatalytic toluene oxidation with nickel-mediated cascaded active units over Ni/Bi2WO6 monolayers
Source: Nat Commun. 2024 May 31;15:4641. doi: 10.1038/s41467-024-49005-6 (PMC11143222; doi:10.1038/s41467-024-49005-6)
Supplement: Supplementary file 1 — Supplementary Information [file 41467_2024_49005_MOESM1_ESM.pdf]

Supplementary information

Photocatalytic toluene oxidation with nickel-mediated cascaded active units over Ni/Bi_2WO_6 monolayers

Yingzhang Shi,[1, 2, 4] Peng Li,[1, 4] Huiling Chen,[1] Zhiwen Wang,[1, 2] Yujie Song,[1, 2] Yu Tang,[1] Sen Lin,[1, *] Zhiyang Yu,[1, *] Ling Wu, [1, *] Jimmy C. Yu[1, 3] and Xianzhi Fu[1]

[1]State Key Laboratory of Photocatalysis on Energy and Environment, Fuzhou University, Fuzhou, Fujian 350116, China
[2]School of Chemistry and Chemical Engineering, Hainan University, Haikou, Hainan 570228, China
[3]Department of Chemistry, The Chinese University of Hong Kong, Shatin, New Territories, Hong Kong, China
[4]These authors contributed equally to this work.

E-mail: slin@fzu.edu.cn; yuzyemlab@fzu.edu.cn; wuling@fzu.edu.cn

Table of Contents

Supplementary Methods....................4

Supplementary Figures....................8

Supplementary Fig. 1 XRD patterns of the prepared BWO, 0.9 Ni/BWO, 1.8 Ni/BWO, 2.6 Ni/BWO....................8

Supplementary Fig. 2 SEM images of the prepared BWO (a), 0.9 Ni/BWO (b), 1.8 Ni/BWO (c), 2.6 Ni/BWO (d)....................8

Supplementary Fig. 3 AFM image of the prepared 1.8 Ni/BWO (a) and its height profiles (b), AFM image of the prepared BWO (c) and height profiles (d)....................9

Supplementary Fig. 4 TEM (a) and HRTEM (b) images and Element mapping images of BWO: Bi (c), O (d), W (e)....................10

Supplementary Fig. 5 BET test results for the prepared samples. BWO (a), 0.9Ni/BWO (b), 1.8 Ni/BWO (c), 2.6 Ni/BWO (d)....................10

Supplementary Fig. 6 A 1.8Ni/BWO single crystal image recorded from the 1.8Ni/BWO sample (a), The corresponding axis (b), Atomic-resolution HAADF images (c) and the corresponding atomic scale information, purple: Bi atoms, blue: W atoms and red: O atoms (d). 11

Supplementary Fig. 7 XPS spectra of the prepared samples. O 1*s* (a), Ni 2*p* (b). Ni 2*p* of 1.8 Ni/BWO after eatching surface Bi and Mo atoms (c)....................11

Supplementary Fig. 8 XPS spectra of the Ni/BWO surface. W 4*f* (a), Bi 4*f* (b), O 1*s* (c), Ni 2*p* (d)....................12

Supplementary Fig. 9 W L_3-edge X-ray absorption fine structure (XAFS) measurements for BWO and 1.8 Ni/BWO: (a) The X-ray absorption near edge structure (XANES) spectra. (b) W L_3-edge extended XAFS (EXAFS) oscillation function $k^2\chi(k)$....................13

Supplementary Fig. 10 The optimized (010) structure model of BWO and Ni/BWO. Side view: (a) optimized structure of BWO, (b) BWO containing Ov, (c) optimized structure of Ni/BWO, (d) Ni/BWO containing Ov; and (e-h) is the top view corresponding to (a-d). The oxygen vacancy is located in the black circle. 14

Supplementary Fig. 11 The optimized (001) structure model of BWO and Ni/BWO. Side view: (a) optimized structure of BWO, (b) BWO containing Ov, (c) optimized structure of Ni/BWO, (d) Ni/BWO containing Ov; and (e-h) is the top view corresponding to (a-d). The potential oxygen vacancy is located in the black circle. 15

Supplementary Fig. 12 The electron density of Bi atoms analysis around the O_V. The Bi atoms are circled by blue line and O_V is circled by black line. The table is the corresponding calculated valence state of selected atoms on the simulated structure. 15

Supplementary Fig. 13 UV–vis DRS spectra of the samples (a), the corresponding $(\alpha h\nu)^{1/2}$ versus E_g plot (b), Mott-Schottky plots of BWO (c) and 1.8 Ni/BWO (d). 16

Supplementary Fig. 14 Schematic energy band diagrams of 1.8 Ni/BWO and BWO. 17

Supplementary Fig. 15 The photos of the reaction apparatus. (a) The reaction tube; (b) The reaction device. 18
Supplementary Fig. 16 XRD pattern of the prepared BWO-bulk, BWO-Ov, Ni/BWO-surface and BiOCl. 18
Supplementary Fig. 17 SEM images of the prepared BWO-bulk (a), BWO-Ov (b), Ni/BWO-surface (c) and BiOCl (d). 19
Supplementary Fig. 18 EPR spectra of the prepared BWO-Ov, Ni/BWO-surface and BiOCl. 20
Supplementary Fig. 19 Gas phase (a) analysis for different reaction time from our online GC setup and the corresponding conversion of TL and selectivity of BD (b); reaction condition: 10 mg 1.8 Ni/BWO, 0.1 mmol TL, 1.5 mL acetonitrile, O_2. Liquid phase (c) and gas phase (d) analysis after reaction from our online GC setup. 21
Supplementary Fig. 20 The yield of benzaldehyde at different temperature in the dark over 1.8 Ni/BWO (a). The cycle experiments of the TL oxidation over 1.8Ni/BWO (b). 21
Supplementary Fig. 21 The XRD pattern of 1.8Ni/BWO before and after the reaction (a). Element mapping images (b) and TEM (c, d) of 1.8Ni/BWO after the reaction. 22
Supplementary Fig. 22 Time-dependent in situ DRIFT spectra of 1.8 Ni/BWO in a toluene and O_2 atmosphere under visible light irradiation. 23
Supplementary Fig. 23 The optimized structures of chemisorbed O_2 (a) and lattice O (b) on Ni/BWO. The lattice O transfer mechanism with the corresponding energies (c). 24
Supplementary Fig. 24 In situ EPR spectra of a carbon-centred radical in toluene solution without O_2 using PBN as spin-trapping agent (a) and using DMPO as spin-trapping agent to detect superoxide radical (b), The conversion rate of toluene in quenching different active species (c). 25
Supplementary Fig. 25 Nyquist impedance plots (a), photocurrent response (b), photoluminescence spectra (c) and time-resolved photoluminescence spectra (d) of the prepared samples. 27
Supplementary Fig. 26 The specific optimized adsorption structures of the C-H bond in toluene on different surface sites (a). The stable configuration of C_7H_7 intermediate adsorbed on Ni/BWO-(001): Green H atom is from the deprotonation of toluene (b). The stable configuration of benzaldehyde adsorbed on Ni/BWO-(001) and BWO-(001) with the desorption energy (c): the bigger desorption energy indicates the more difficult desorption of benzaldehyde. 28
Supplementary Fig. 27 Possible mechanism for the oxidation of toluene based on semiconductor energy band theory and photo-induced active radical. 29
Supplementary Tables 30
Supplementary Table 1 The actual mass fraction of Ni calculated by ICP-MS results. 30
Supplementary Table 2 The fraction of W^{5+} and W^{6+} in the prepared samples. 31
Supplementary Table 3 The fraction of O species in the prepared samples. 32
Supplementary Table 4 The fitting results of W L_3-edge XAFS spectra 32

Supplementary Table 5 Apparent quantum efficiency (AQE) of the prepared samples for toluene oxidation....33
Supplementary Table 6 photocatalytic oxidation of toluene derivatives....34
Supplementary Table 7 The previous studies for the photocatalytic TL oxidation. 35
Supplementary Table 8 The adsorption energy for O_2 on unsaturated Bi, Ni and W sites. 36
Supplementary Table 9 The comparison of vibration frequency and corresponding force constant of the C–H bonds in TL molecules chemisorbed on the samples. The force constants (k) are calculated by molecular vibration equation $\upsilon = 1303(k/M)^{1/2}$. υ is vibration frequency from FTIR spectrum. M represents convert mass of C atom and H atom. 36
Supplementary Table 10 The Bader charge of spatially separated Bi and O atoms in Ni/BWO-(001) and Ni-OH/BWO-(001) structure modes. 37
Supplementary Reference 38

Supplementary Methods

Materials

$Bi(NO_3)_3$•5 H_2O (≥99%), Na_2WO_4•$2H_2O$ (≥99.5%), $Ni(NO_3)_2$•$6H_2O$, toluene, benzyl alcohol, benzaldehyde, benzoic acid, nitric acid, methanol, NaCl, mannitol, polyvinylpyrrolidone K30 are purchased from Sinopharm Chemical Reagent Co. (SCRC).

Preparation of Bi_2WO_6 nanosheets with O vacancy (BWO-Ov)

BWO-Ov was prepared via a simple heat treatment method. 200 mg of BWO was treated under vacuum at 200 ℃ for 2 h. The heating rate was set as 10 °C /min.

Loading 1.8 w% Ni on Bi_2WO_6 nanosheets (Ni/BWO-surface)

Ni/BWO-surface was prepared via a photodeposition method. $Ni(NO_3)_2$•$6H_2O$ and BWO were added to 50 mL deionized water in exact proportion under stirring. Then the mixture was bubbled using N_2 to remove O_2 and 5 mL methanol was added as hole sacrifice agent. It was irradiated under

a 300 W Xenon lamp (Beijing Perfectlight Technology Co. Ltd., PLS-SXE300D) for 4 h. The result product was washed with deionized water and dried at 60 ℃ for 24 h. The obtained powder was further heated at 200 ℃ in O_2 atmosphere. The final sample was named as Ni/BWO-surface.

Preparation of BiOCl nanosheets with O vacancy (BiOCl)

BiOCl was prepared via the method reported in our previous studies.[1] 0.002 mol of $Bi(NO_3)_3$•5 H_2O and 0.800 g of PVPk30 were added into 50 mL of 0.1 M mannitol solution under stirring. Then, 10 mL of saturated NaCl solution was added into the mixed solution. The mixture was transferred into a 100 mL Teflon-lined stainless-steel autoclave after stirring for 10 min and heated at 160 ℃ for 5 h. The precipitates were washed with ethanol and deionized water. Finally, the products were dried at 60 ℃ for 6 h.

In situ DRIFT and EPR test

The FT-IR spectra of the powder samples and the time-dependent in situ diffuse reflectance infrared Fourier-transform (DRIFT) spectra were recorded on a NICOLET IS50 Fourier transform infrared (FT-IR) spectrometer at a resolution of 4 cm^{-1}, undergoing a total of 64 scans. Before collecting the in situ DRIFTS spectra, the samples were treated at 150 ℃ for 2 h in N_2 atmosphere. Then pure O_2 (99.99%) was continuously introduced into the sample chamber. The time-dependent in situ DRIFT spectra for adsorbing O_2 in the dark were recorded. For the in-situ characterization of the photocatalytic toluene oxidation, pure O_2 (99.99%) saturated with toluene vapor was continuously introduced into the sample chamber. The time-dependent in situ DRIFT spectra for adsorbing toluene in the dark were recorded. Next, the sample was irradiated using a 300-W Xe lamp. The time-dependent in situ DRIFT spectra under visible light irradiation were recorded.

EPR spectra of the powder samples were recorded on a Brucker A300 spectrometer. The in situ EPR spectra for detecting carbon central radical were carried out under visible-lightirradiation (λ> 400 nm). 10 mg of the prepared sample, 0.1 mmol of N-tert-Butyl-α-phenylnitrone (PBN), 2 mL of toluene were mixed in a quartz reactor. N_2 gas was passed through the solution to remove O_2. Then, the mixture was collected by a capillary and transferred to a closed EPR tube with N_2 and then the in situ EPR spectra of the samples were recorded. The in situ EPR spectra for detecting $\bullet O_2^-$ radical were carried out under the same method, but the trapping agent was 5,5-dimethyl-1-pyrroline-N-oxide (DMPO) and the atmosphere was O_2.

Apparent quantum efficiency of the conversion of TL and turnover frequency

The apparent quantum efficiency (AQE) for the conversion of TL was measured using a 300 W Xenon lamp with a 420 nm band pass filter.[2] The total irradiance was 15.0 mW cm^{-2}. The irradiation area was controlled as 4 cm^2. Depending on the amount of converted TL by the photocatalytic reaction in 6 hours, and the AQY was calculated as follow:

$$\eta_{AQY} = \frac{N_e}{N_p} \times 100\% = \frac{2 \times M \times N_A}{\frac{Etotal}{Ephoton}} \times 100\% = \frac{2 \times M \times N_A}{\frac{S \times P \times t}{\hbar \times \frac{c}{\lambda}}} \times 100\%$$

$$= \frac{2 \times M \times N_A \times \hbar \times c}{S \times P \times t \times \lambda} \times 100\%$$

Where, M is the molar mumber of transformed TL (mol), N_A represents Avogadro constant (6.022×1023 mol^{-1}), $\hbar$ represents the Planck constant (6.626×10^{-34} J·s), c represents the speed of light (3×10^8 m s^{-1}), S represents the irradiation area (cm^2), P represents the intensity of irradiation light (W cm^{-2}), t represents the reaction time (s), λ represents the wavelength of the monochromatic light (nm).

Turnover frequency (TOF) is calculated according to the formulas in previous study using the moles of the catalyst.[3]

TOF = moles of consumed toluene / (moles of catalyst × reaction time).

Supplementary Figures

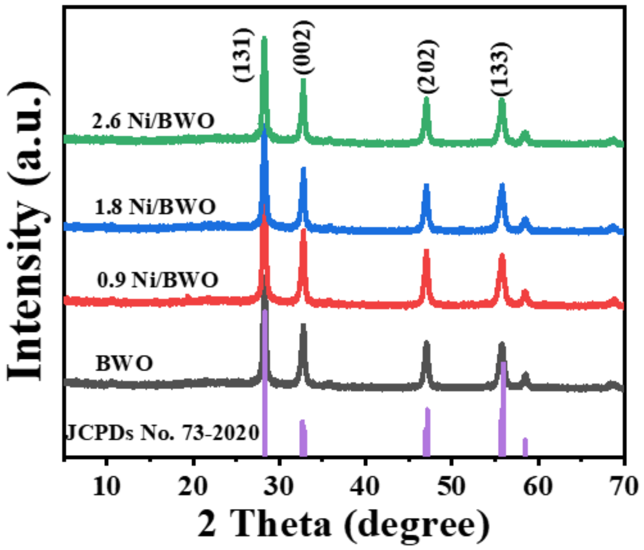

Supplementary Fig. 1 XRD patterns of the prepared BWO, 0.9 Ni/BWO, 1.8 Ni/BWO, 2.6 Ni/BWO.

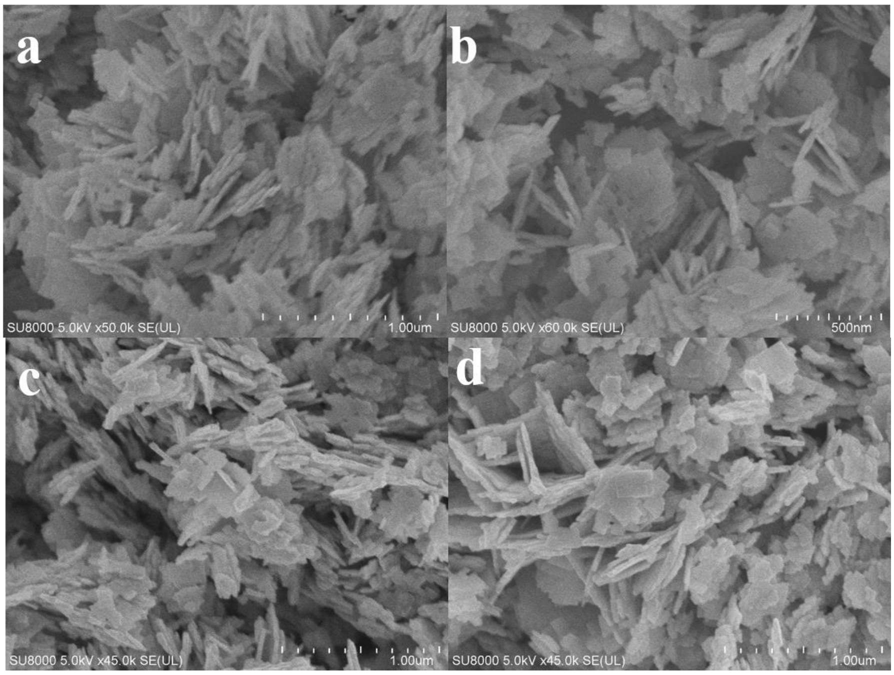

Supplementary Fig. 2 SEM images of the prepared BWO (a), 0.9 Ni/BWO (b), 1.8 Ni/BWO (c), 2.6 Ni/BWO (d).

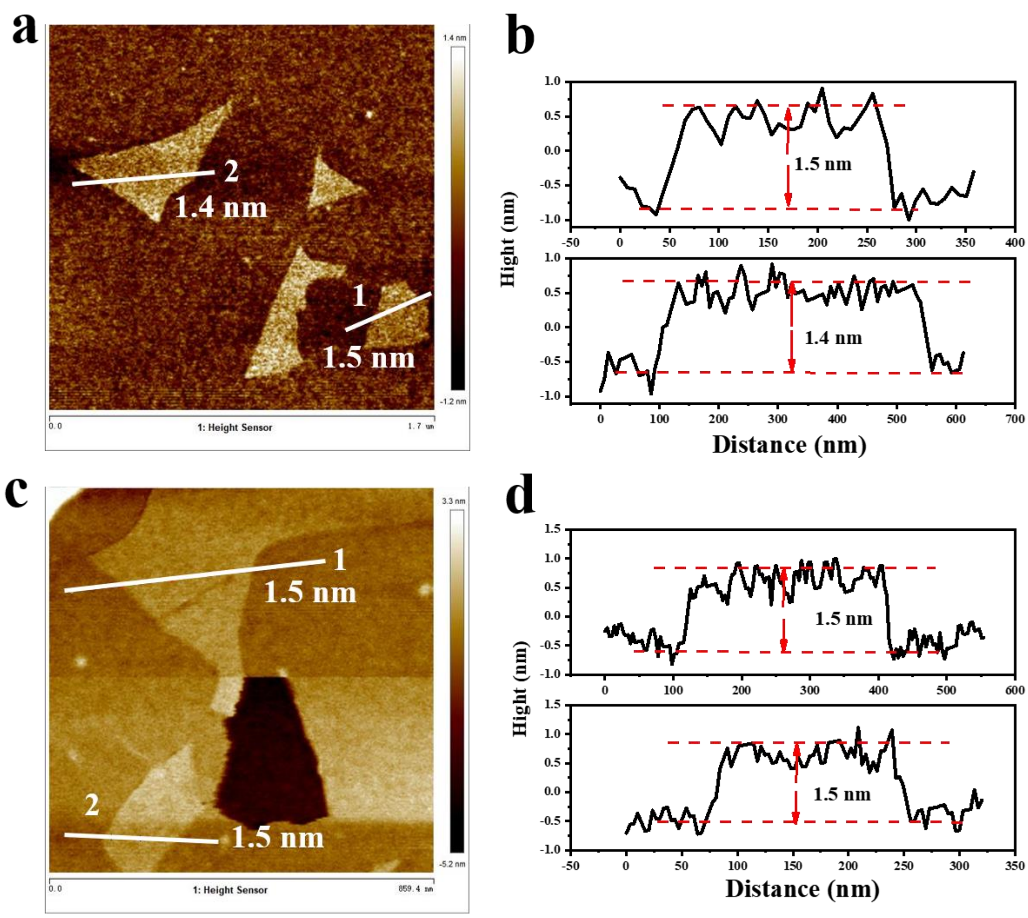

Supplementary Fig. 3 AFM image of the prepared 1.8 Ni/BWO (a) and its height profiles (b), AFM image of the prepared BWO (c) and height profiles (d).

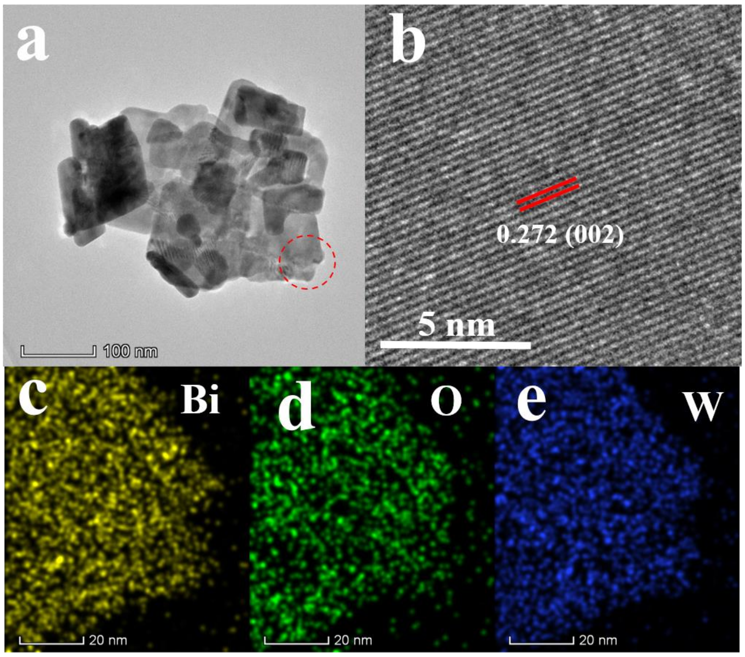

Supplementary Fig. 4 TEM (a) and HRTEM (b) images and Element mapping images of BWO: Bi (c), O (d), W (e).

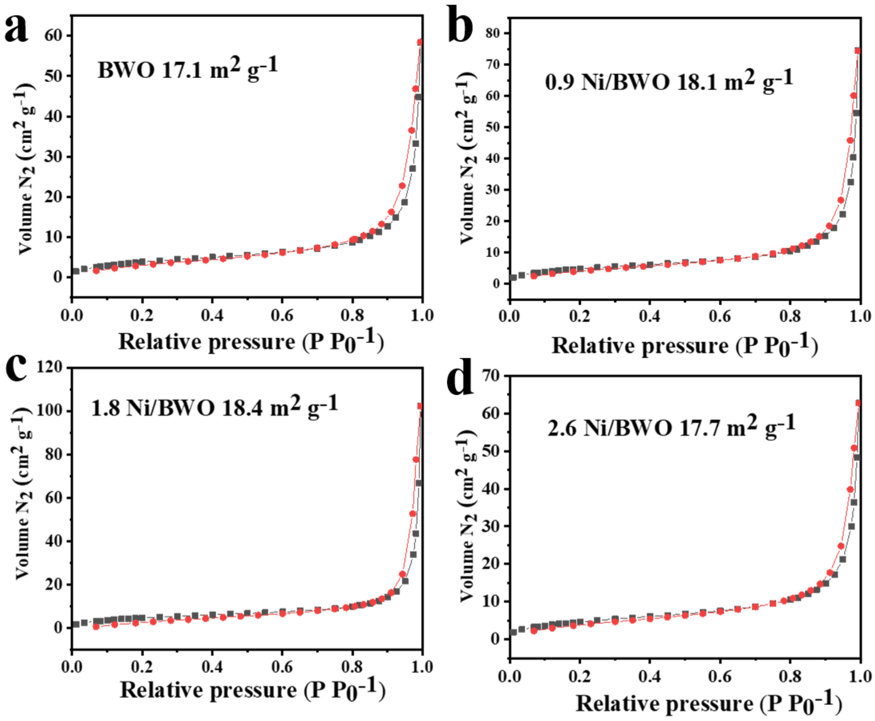

Supplementary Fig. 5 BET test results for the prepared samples. BWO (a), 0.9Ni/BWO (b), 1.8 Ni/BWO (c), 2.6 Ni/BWO (d).

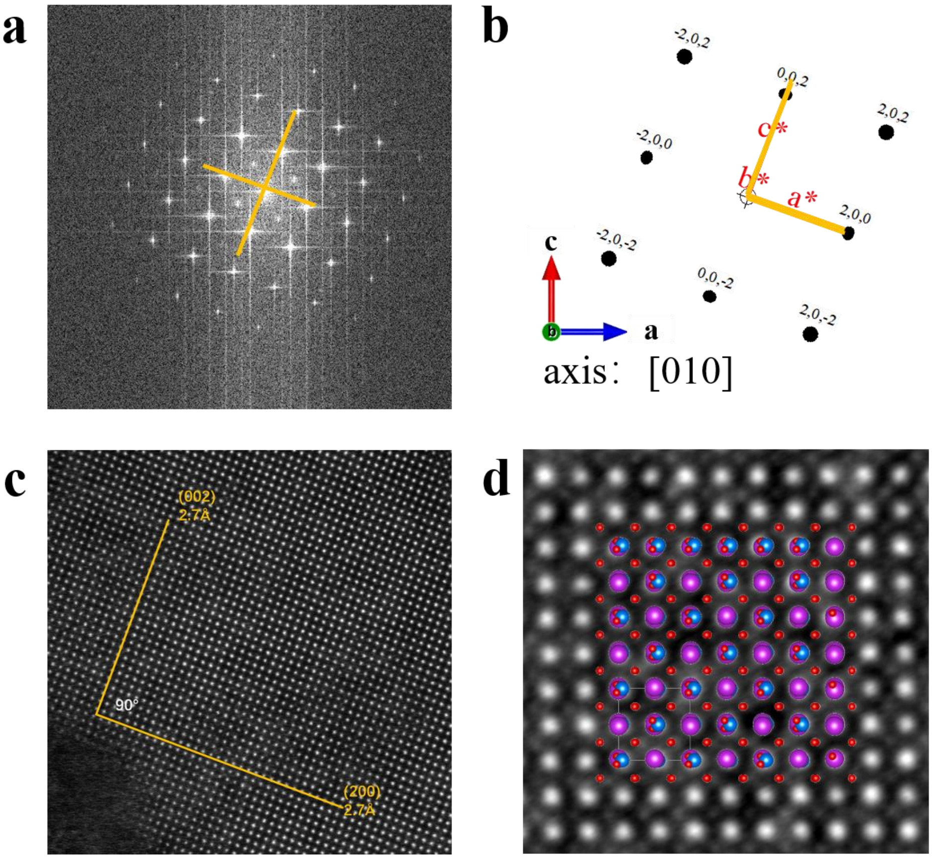

Supplementary Fig. 6 A 1.8Ni/BWO single crystal image recorded from the 1.8Ni/BWO sample (a), The corresponding axis (b), Atomic-resolution HAADF images (c) and the corresponding atomic scale information, purple: Bi atoms, blue: W atoms and red: O atoms (d).

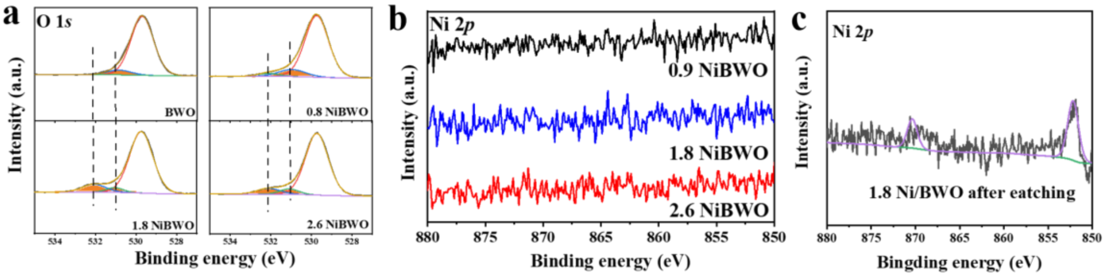

Supplementary Fig. 7 XPS spectra of the prepared samples. O 1*s* (a), Ni 2*p* (b). Ni 2*p* of 1.8 Ni/BWO after eatching surface Bi and Mo atoms (c).

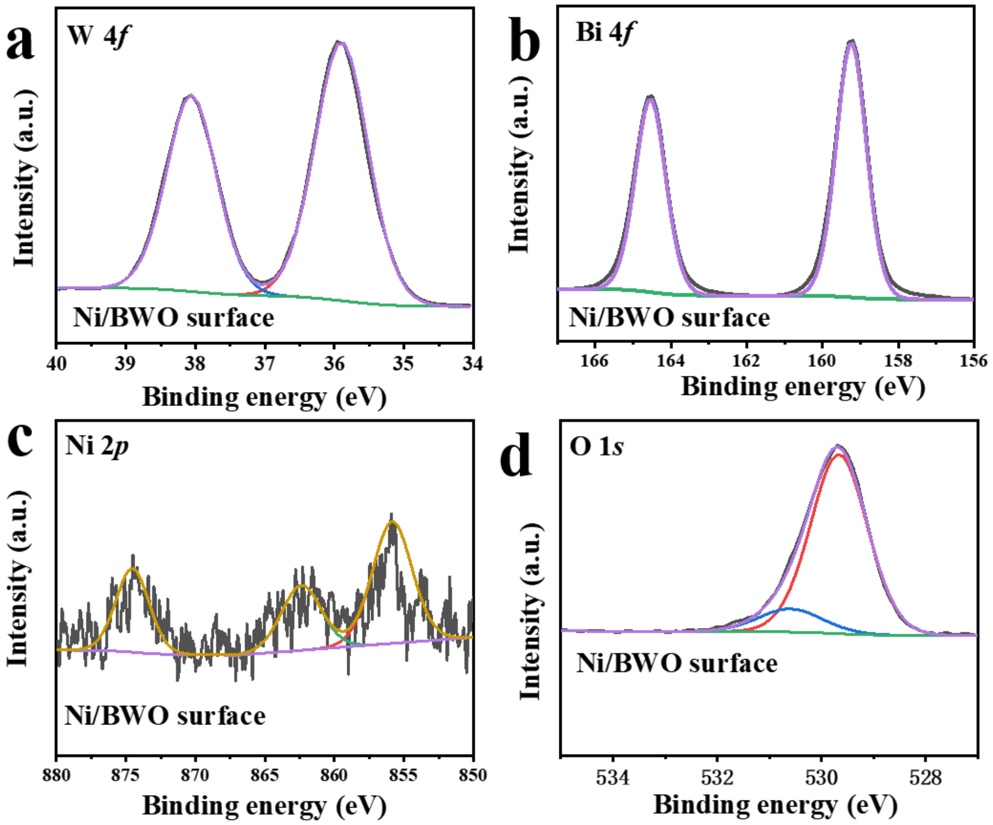

Supplementary Fig. 8 XPS spectra of the Ni/BWO surface. W 4*f* (a), Bi 4*f* (b), O 1*s* (c), Ni 2*p* (d).

When the Ni is loaded on the surface of BWO by photodeposition, Ni^{2+} XPS signal of Ni/BWO-surface can be detected. It also suggests that Ni is doped into the crystal lattice of 1.8 Ni/BWO rather than surface. Thus, no signals are detected in Figure S6c. The signal of Ni^{2+} is weak, which is due to the small content of Ni. In addition, no W^{5+} is fitted, indicating that loading Ni on surface of BWO would not cause the obvious microstructure change.

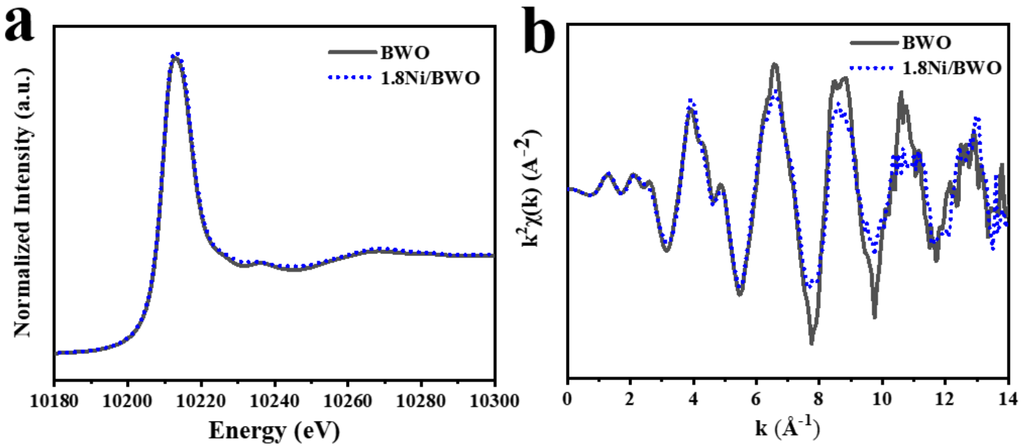

Supplementary Fig. 9 W L_3-edge X-ray absorption fine structure (XAFS) measurements for BWO and 1.8 Ni/BWO: (a) The X-ray absorption near edge structure (XANES) spectra. (b) W L_3-edge extended XAFS (EXAFS) oscillation function $k^2\chi(k)$.

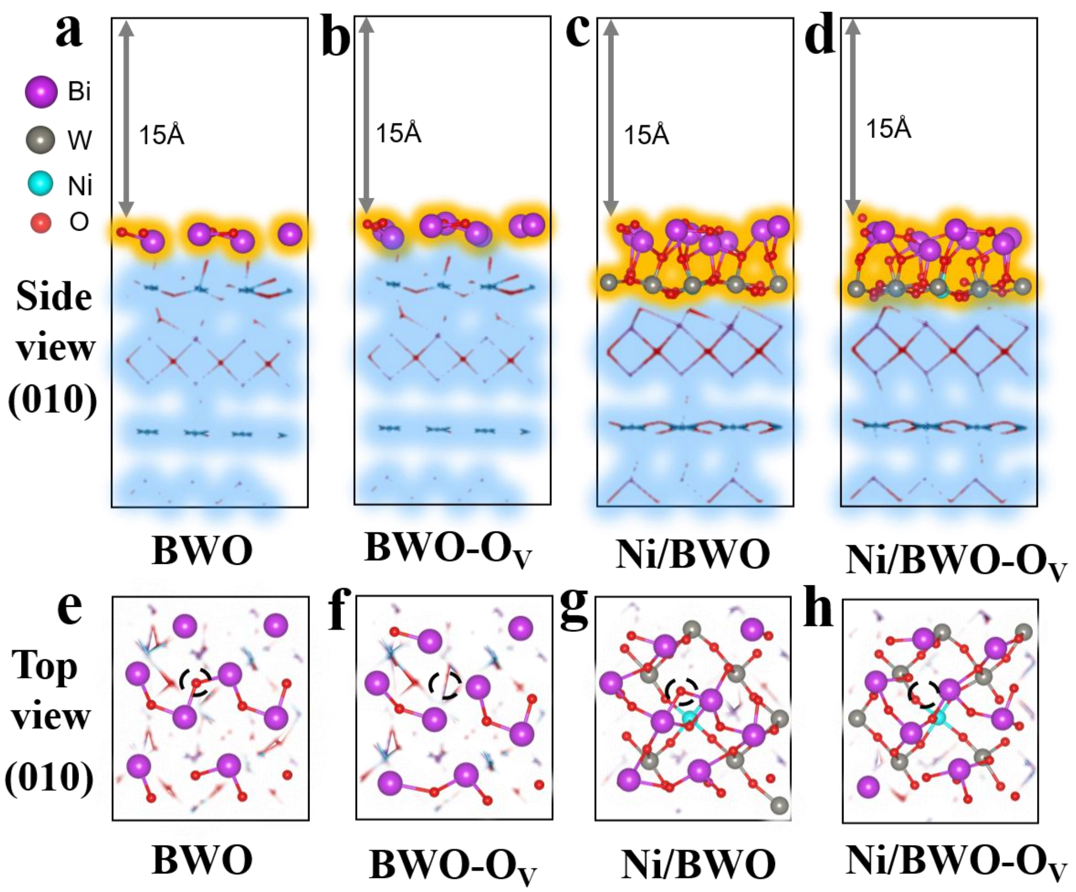

Supplementary Fig. 10 The optimized (010) structure model of BWO and Ni/BWO. Side view: (a) optimized structure of BWO, (b) BWO containing Ov, (c) optimized structure of Ni/BWO, (d) Ni/BWO containing Ov; and (e-h) is the top view corresponding to (a-d). The oxygen vacancy is located in the black circle.

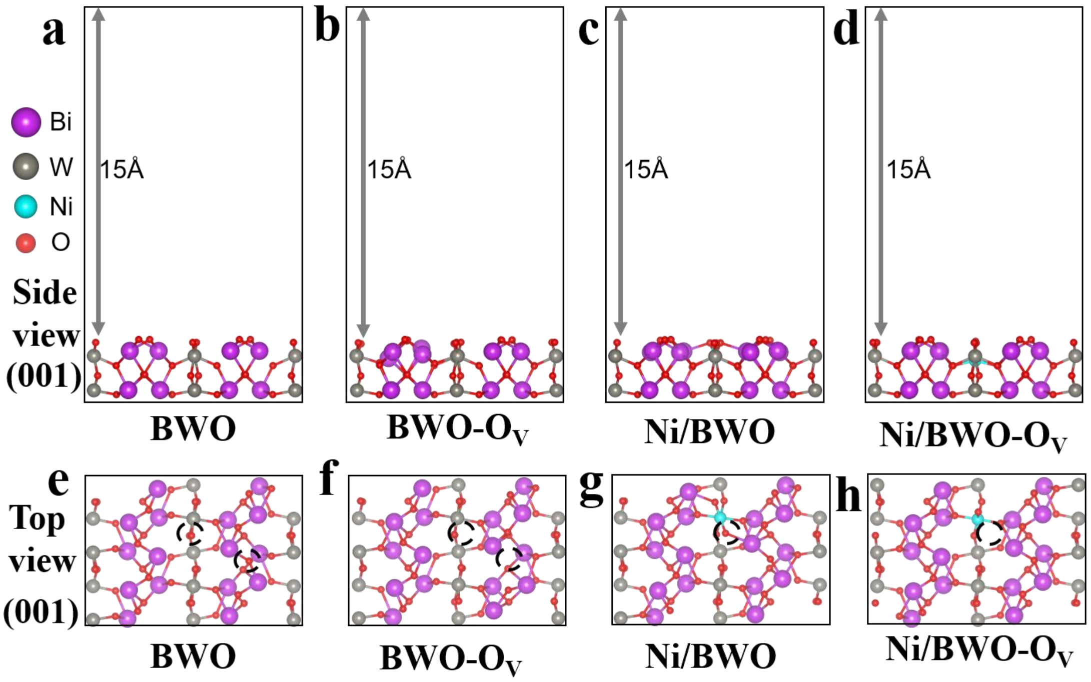

Supplementary Fig. 11 The optimized (001) structure model of BWO and Ni/BWO. Side view: (a) optimized structure of BWO, (b) BWO containing Ov, (c) optimized structure of Ni/BWO, (d) Ni/BWO containing Ov; and (e-h) is the top view corresponding to (a-d). The potential oxygen vacancy is located in the black circle.

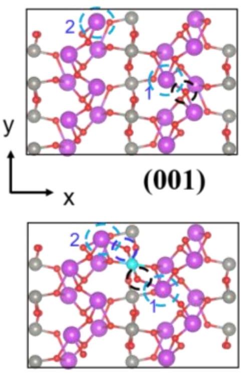

BWO(001)-Ov		BWO(010)-Ov	
Bi1	1.978	Bi1	1.469
Bi2	1.903	Bi2	1.329
**Ni/BWO(001)-Ov**		**Ni/BWO(010)-Ov**	
Bi1	1.984	Bi1	1.857
Bi2	2.051	Bi2	1.624
Ni	1.258	Ni	1.335

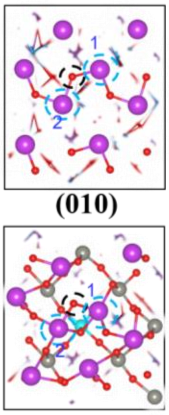

Supplementary Fig. 12 The electron density of Bi atoms analysis around the O$_V$. The Bi atoms are circled by blue line and O$_V$ is circled by black line. The table is the corresponding calculated valence state of selected atoms on the simulated structure.

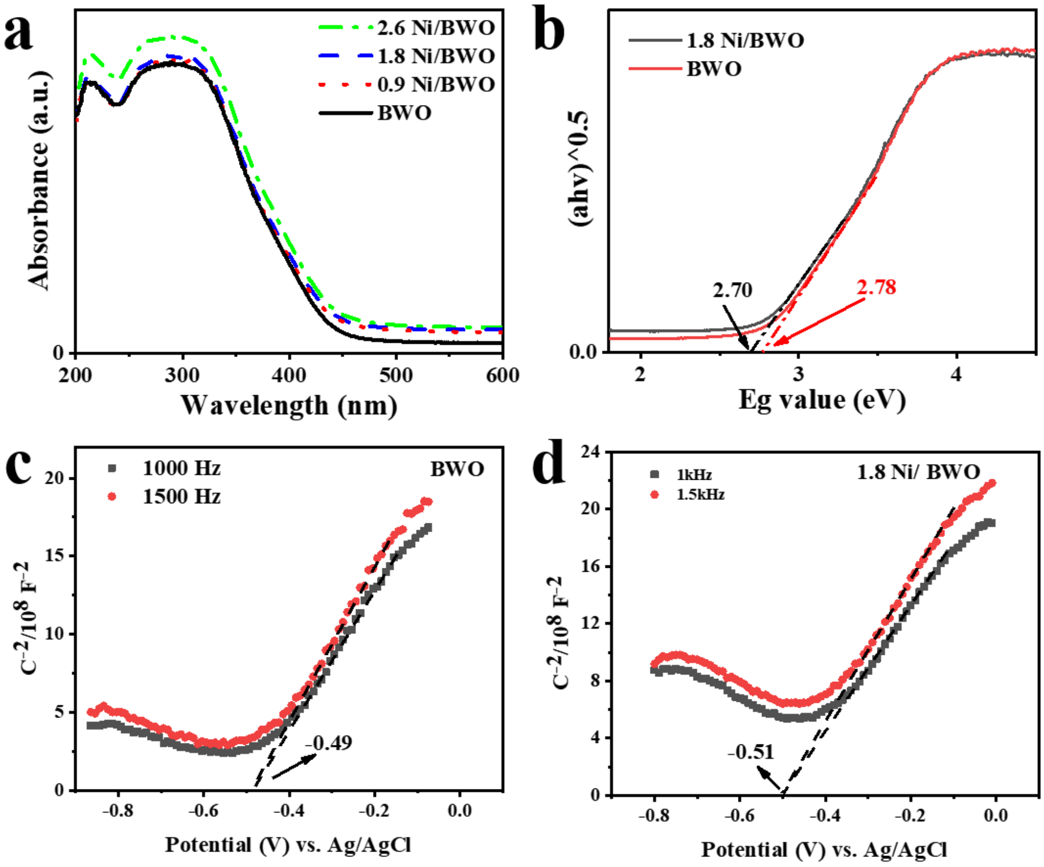

Supplementary Fig. 13 UV–vis DRS spectra of the samples (a), the corresponding $(\alpha hv)^{1/2}$ versus E_g plot (b), Mott-Schottky plots of BWO (c) and 1.8 Ni/BWO (d).

The light absorption properties of the samples were investigated by UV–vis diffuse reflectance spectra (UV-vis DRS). Supplementary Fig. 13a exhibits that the UV-vis DRS of BWO and Ni/BWO also have similar absorption curves. The slight red shift of Ni/BWO is observed due to the weak metal-to-metal charge transfer (MMCT) led by W-O-Ni bonds. The band gap values of 1.8 Ni/BWO and BWO (Supplementary Fig. 13b) are 2.70 and 2.78 eV, respectively. The Mott-Schottky curves of BWO and 1.8 Ni/BWO (Supplementary Fig. 8c, d) are also analyzed to determine the CB potentials of BWO and 1.8 Ni/BWO. It is obvious that the slope of Mott-Schottky curves at different frequencies are positive, suggesting that BWO and 1.8 Ni/BWO are both the n-type semiconductor. The flat band potential in Supplementary Fig. 8c and

Supplementary Fig. 8d are -0.49 and -0.51 V vs. Ag/AgCl, respectively. It is reported that the CB potential of n-type semiconductor is approximately equal to the flat band potential. Meanwhile, there is an equation between the normal hydrogen electrode potential (NHE) and the Ag/AgCl electrode potential as follow: E (NHE) = E (Ag/AgCl) - E^{θ} + 0.059 pH. Where E^{θ} (Ag/AgCl, at pH =7) = 0.197 V.[4] Therefore, the CB potentials of BWO and 1.8 Ni/BWO are -0.31 and -0.29 V vs. NHE, respectively. The energy band value (Eg) of BWO and 1.8 Ni/BWO (Supplementary Fig. 8b) are 2.78 and 2.7 eV, calculating the VB potentials of BWO and 1.8 Ni/BWO are 2.49 and 2.39 V vs. NHE, respectively. Shown in Supplementary Fig. 14 is the schematic of the energy band for 1.8 Ni/BWO and BWO. The CB and VB of Bi_2WO_6 are constituted via W-5*d* and Bi-6*s*/O2*p* hybrid orbitals, respectively. The corresponding potentials manifest that 1.8 Ni/BWO and BWO both possess the ability to reduce O_2 to $\bullet O_2^-$ (-0.28 V vs. NHE) and oxidizing TL (2.18 V vs. NHE). This result indicates that the photocatalytic oxidation of TL over Bi_2WO_6 is feasible in terms of thermodynamics.

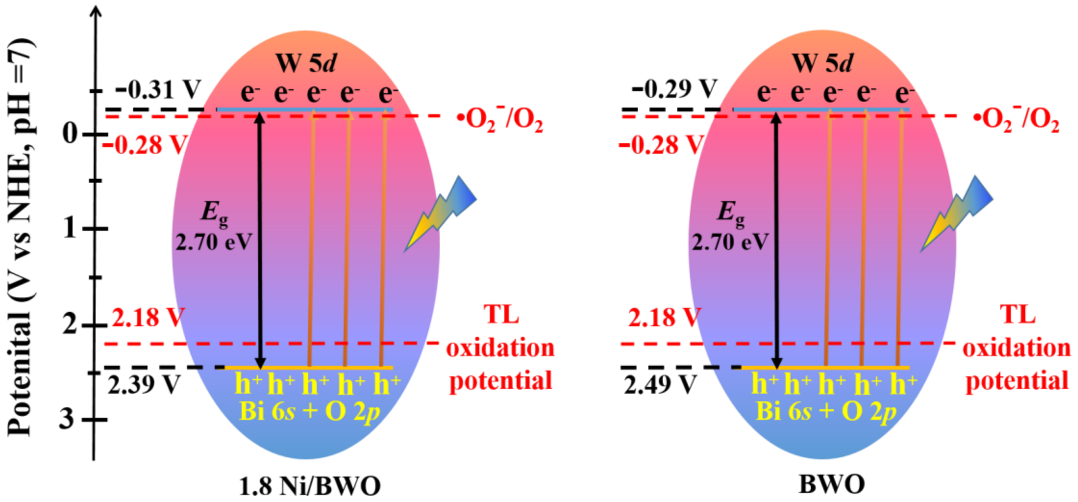

Supplementary Fig. 14 Schematic energy band diagrams of 1.8 Ni/BWO and BWO.

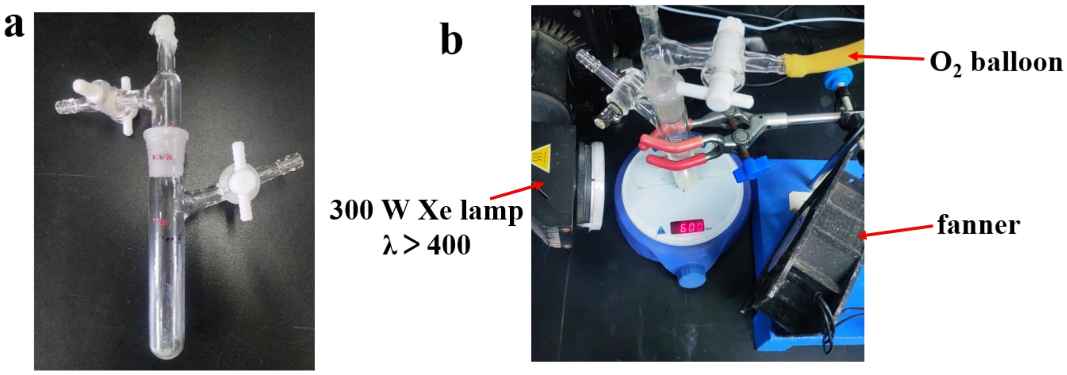

Supplementary Fig. 15 The photos of the reaction apparatus. (a) The reaction tube; (b) The reaction device.

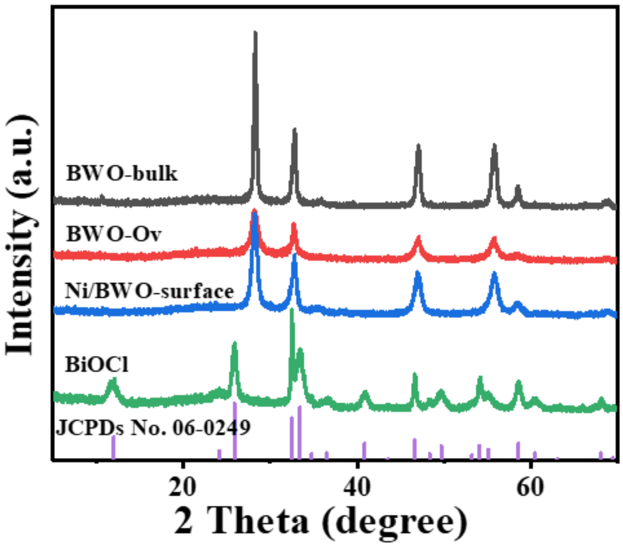

Supplementary Fig. 16 XRD pattern of the prepared BWO-bulk, BWO-Ov, Ni/BWO-surface and BiOCl.

In order to illustrate the important roles of the FLPs, unsaturated W and O_V induced by Ni dopant for TL oxidation, bulk Bi_2WO_6 (BWO-bulk), Bi_2WO_6 nanosheet with O_V (BWO-Ov), Bi_2WO_6 nanosheet with surface Ni (Ni/BWO-surface) and BiOCl with O_V were prepared for comparison. BWO-bulk is prepared to highlight the advantage of monolayer nanosheets. BWO-Ov is used to study the role of Ov, unsaturated W and Bi atoms. Ni atoms is loaded on the

surface of BWO nanosheet without the formation of Ov and unsaturated W to study the influence of Ni. In addition, BiOCl with Ov is conducted to illustrate the influence of Ov and unsaturated Bi atoms. As shown in Supplementary Fig. 11, XRD peaks of all the prepared BWO-bulk, BWO-Ov and Ni/BWO-surface samples are well matched with orthorhombic Bi_2WO_6. The peak intensity of BWO-bulk is the strongest, indicating its good crystallinity. After the heat treatment, the peak intensity of BWO-Ov is the lowest, which forebodes the formation of Ov. Moreover, after loading Ni on the surface of BWO, no other peaks are observed. This may be due to the small content of Ni. The XRD pattern of BiOCl is also well match with the with the published data (JCPDS No. 6-0249),[1] confirming its successful preparation.

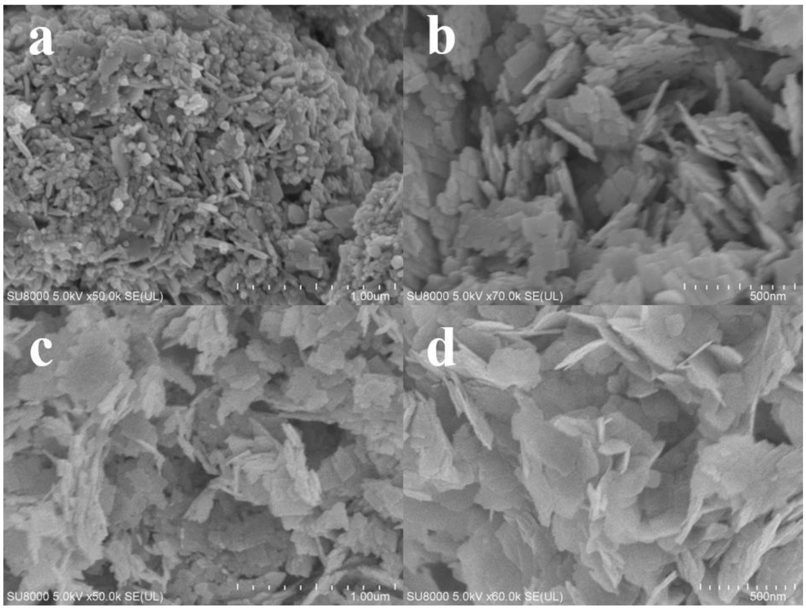

Supplementary Fig. 17 SEM images of the prepared BWO-bulk (a), BWO-Ov (b), Ni/BWO-surface (c) and BiOCl (d).

SEM images of these samples are shown in Supplementary Fig. 12. BWO-bulk shows a morphology of bulk accumulation (Supplementary Fig. 12a). This morphology is disadvantageous for the exposure of surface active sites. After the heat treatment of BWO nanosheet, the nanosheet structure is still remained. Similarly, Ni/BWO-surface and BiOCl also exhibit the nanosheet structure. The morphologies of these samples are well controlled, which is convenient to study

the influence of surface Ov, surface Ni atoms, unsaturated W and FLPs for the photocatalytic TL oxidation. The surface Ov signals of these nanosheets are investigated by EPR test (Supplementary Fig. 13). BWO-Ov and BiOCl both have Ov signals, but no Ov signal is detected for Ni/BWO-surface. It confirms that the Ov is induced after the heat treatment of BWO nanosheet. However, the O_V induced by heat treatment is random and there are hard to create FLPs without Ni dopants during the heat treatment process according the DFT calculation results. To study the influence of Ni, Ni atoms is loaded on the surface of BWO nanosheet via photodeposition. However, this way cannot induce the formation of Ov and unsaturated W. In addition, BiOCl with Ov is conducted to illustrate the influence of Ov and unsaturated Bi atoms.

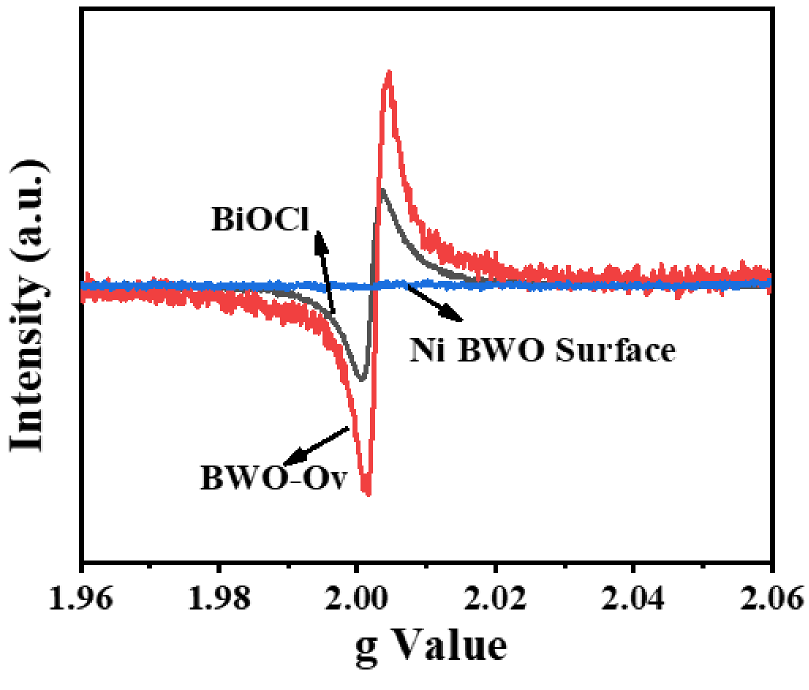

Supplementary Fig. 18 EPR spectra of the prepared BWO-Ov, Ni/BWO-surface and BiOCl.

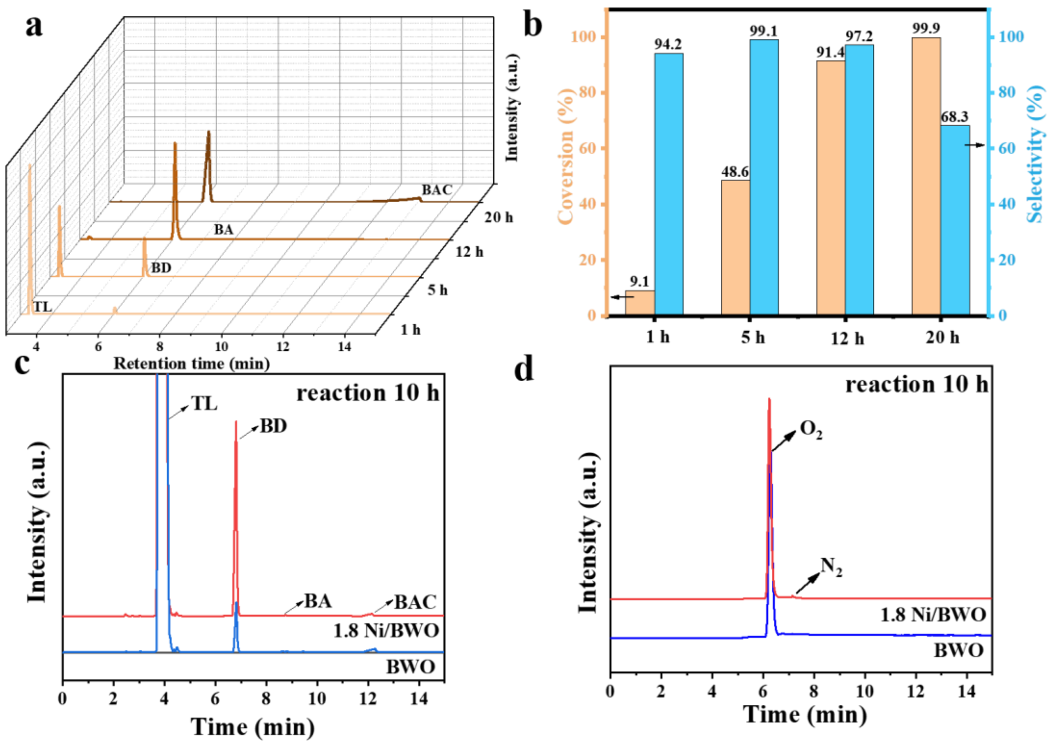

Supplementary Fig. 19 Gas phase (a) analysis for different reaction time from our online GC setup and the corresponding conversion of TL and selectivity of BD (b); reaction condition: 10 mg 1.8 Ni/BWO, 0.1 mmol TL, 1.5 mL acetonitrile, O_2. Liquid phase (c) and gas phase (d) analysis after reaction from our online GC setup.

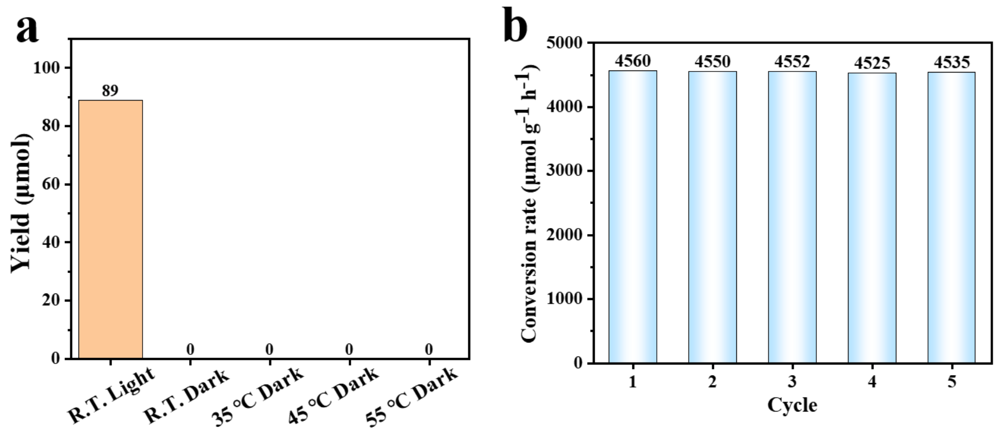

Supplementary Fig. 20 The yield of benzaldehyde at different temperature in the dark over 1.8 Ni/BWO (a). The cycle experiments of the TL oxidation over 1.8Ni/BWO (b).

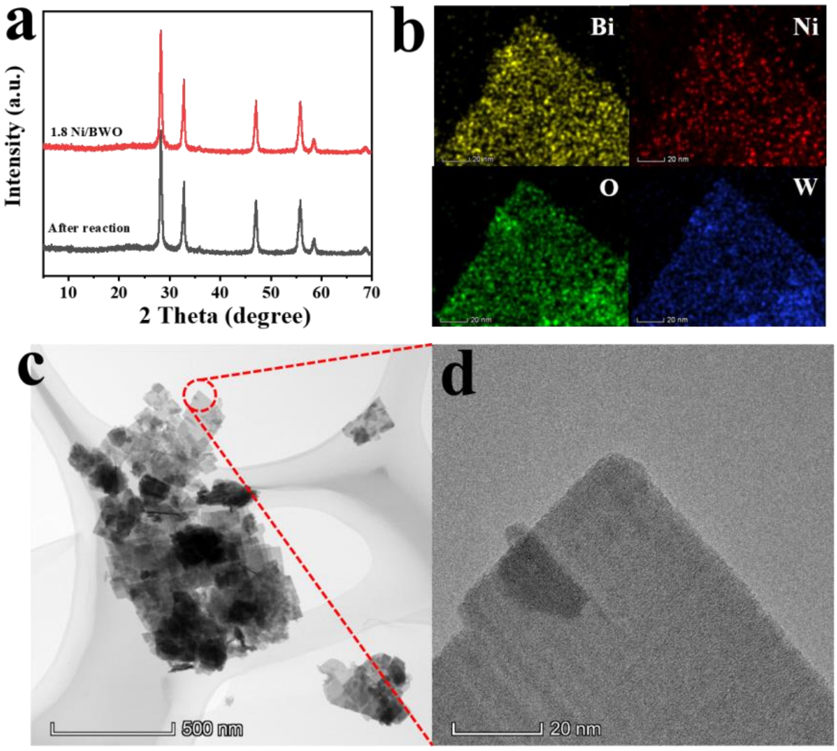

Supplementary Fig. 21 The XRD pattern of 1.8Ni/BWO before and after the reaction (a). Element mapping images (b) and TEM (c, d) of 1.8Ni/BWO after the reaction.

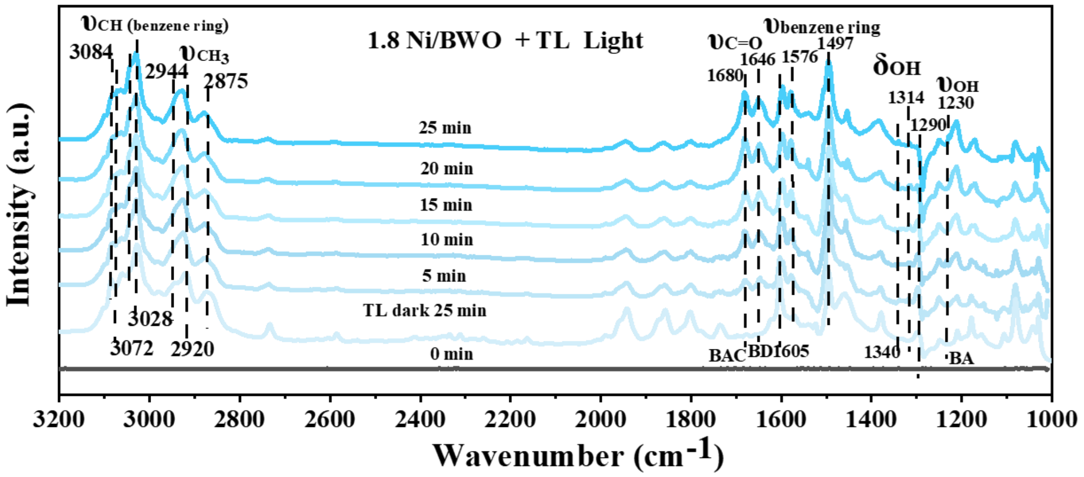

Supplementary Fig. 22 Time-dependent in situ DRIFT spectra of 1.8 Ni/BWO in a toluene and O_2 atmosphere under visible light irradiation.

The characteristic peak of CO_2 in FITR spectra generally locates at 2000-2500 cm^{-1}.[5, 10] From Supplementary Fig. 22, we can see that no characteristic peak of CO_2 is detected as the illumination time increase. It also confirms that the overoxidation of toluene to CO_2 is impossible using 1.8 Ni/BWO as a photocatalyst.

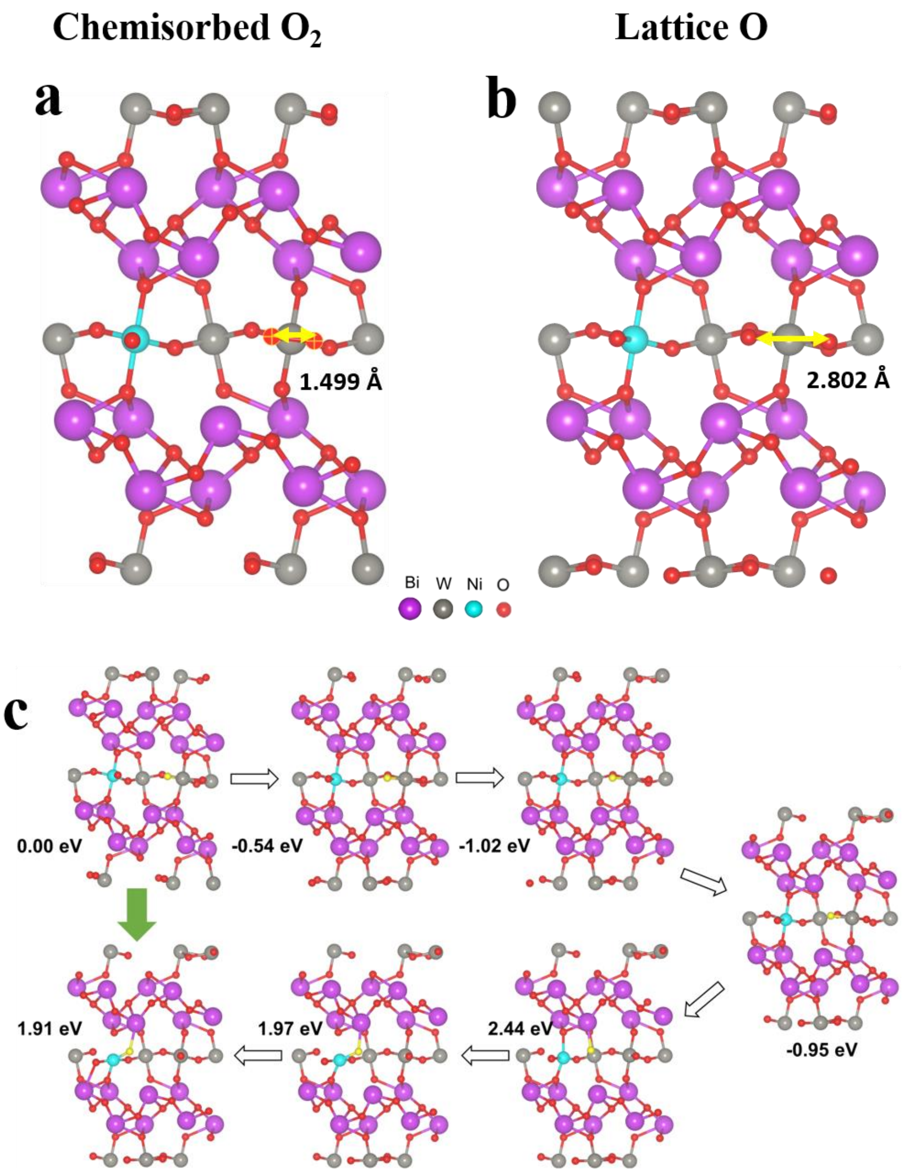

Supplementary Fig. 23 The optimized structures of chemisorbed O_2 (a) and lattice O (b) on Ni/BWO. The lattice O transfer mechanism with the corresponding energies (c).

A comparison was made between the O-O and W-O bond lengths of O_2 adsorbed on the W surface and lattice oxygen atoms coordinated with W, revealing that the lattice O is in a different chemical state from that of O in O_2. The lattice O-O bond length is 2.802 Å and the W-O bond length is 1.765 Å (Supplementary Fig. 23a). The adsorbed O_2, on the other hand, has an O-O bond length of 1.499 Å and a W-O bond length of 1.925 Å (Supplementary Fig. 23b). This indicates that the interaction between lattice O and W is much

stronger than the W-O interaction in adsorbed oxygen species, making the migration process of lattice O may be more difficult. The activation energy for lattice O (6-coordinated O with W) to migrate to a 5-coordinated Ni atom was calculated to be 2.44 eV (with a reaction energy of 1.91 eV). (Supplementary Fig. 23c) This is 2.1 eV higher than the oxygen migration barrier in adsorbed oxygen species. Therefore, the ^{16}O product in isotope experiments is very likely derived from the pre-adsorbed O_2 in the air. The lattice O involved mechanism is unlikely to occur in our system.

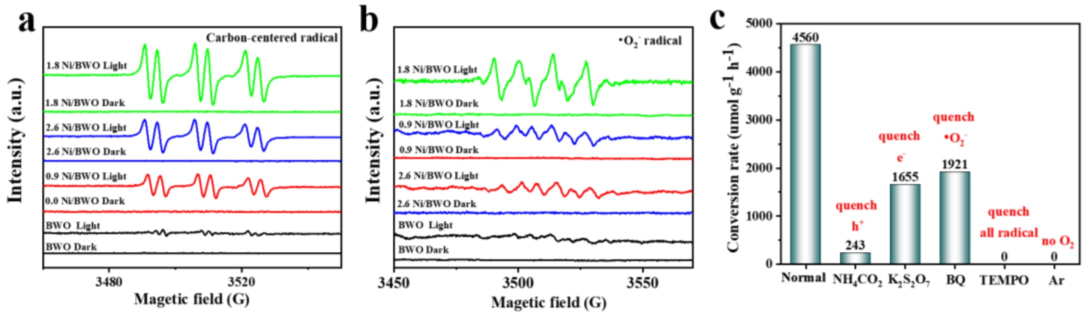

Supplementary Fig. 24 In situ EPR spectra of a carbon-centred radical in toluene solution without O_2 using PBN as spin-trapping agent (a) and using DMPO as spin-trapping agent to detect superoxide radical (b), The conversion rate of toluene in quenching different active species (c).

In situ EPR spectra (Supplementary Fig. 24a) show that when the trapping agent is N-tert-Butyl-α-phenylnitrone (PBN), all prepared samples exhibit the carbon-centered radical signals ($^{*}C_7H_7$) due to the deprotonation of TL by photogenerated holes. Meanwhile, when using 5,5-dimethyl-1-pyrroline-N-oxide (DMPO) as a trapping agent, $•O_2^-$ radical signals are detected (Supplementary Fig. 24b). No signals are detected in the dark, indicating the necessary condition of the visible light irradiation. 1.8 Ni/BWO shows the strongest EPR signal which is consistent with its best performance in TL oxidation. One reason for this is that the O_V favors the separation of photogenerated carriers (Supplementary Fig. 25). Another important reason is that the unsaturated W atoms and FLPs in 1.8 Ni/BWO enhance the activation

of O_2 and TL molecules and establish the migration routes between the adsorbed substrate and the photogenerated carriers, facilitating the deprotonation of TL to a carbon-centered radical and the reduction of O_2 to $•O_2^-$ radical, easily.

In addition, the free radical quenching experiments further explain the role of these active species (Supplementary Fig. 24c). When NH_4COOH is used to quench photogenerated h^+, the conversion rate of TL is significantly reduced to 243 μmol g^{-1} h^{-1}, indicating that the deprotonation of TL by photogenerated holes is the key step for the oxidation of TL. Due to the similar valence band potential of BWO (2.49 V vs. NHE) and 1.8 Ni/BWO (2.39 V vs. NHE) (Supplementary Fig. 13), they all meet the oxidation potential of TL (2.18 V vs. NHE). However, BWO produces the weakest carbon-centered radical signal. This reflects the importance of the adsorption and activation of TL on the FLPs. Moreover, there is no TL to be converted without O_2, as the oxidation of TL requires O_2 as an oxygen source. When the photogenerated electrons and $•O_2^-$ radicals are quenched by $K_2S_2O_7$ and BQ, the conversion rate of TL is 1655 and 1921 μmol g^{-1} h^{-1}, respectively. This result indicates that $•O_2^-$ radicals are considered as one of the active O species in the subsequent oxidation process and another O transfer pathway also exists.

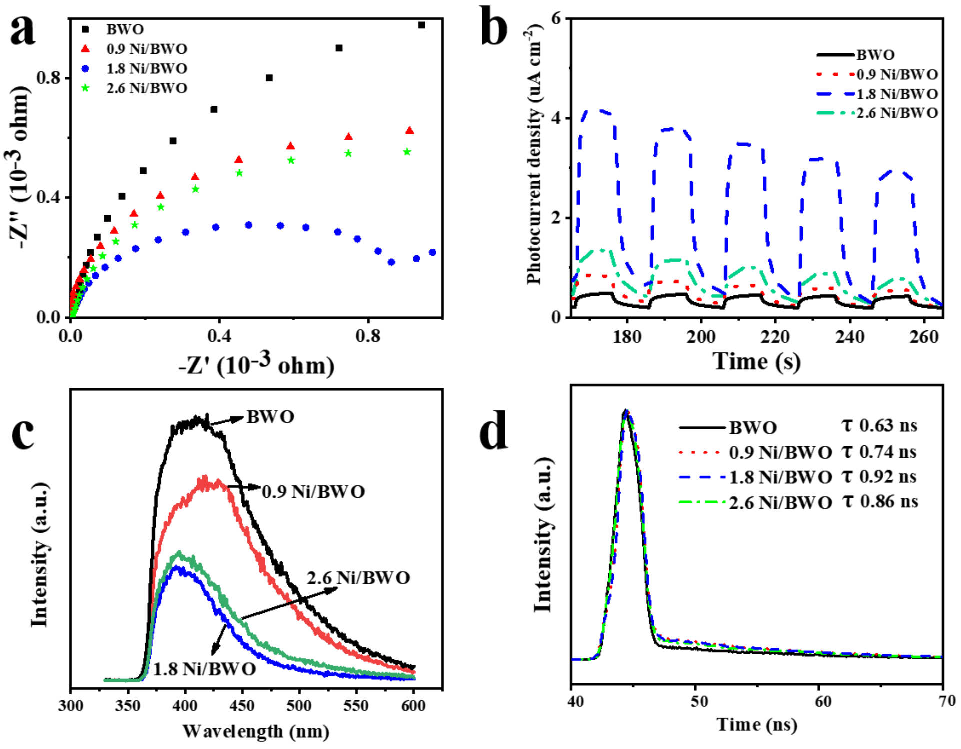

Supplementary Fig. 25 Nyquist impedance plots (a), photocurrent response (b), photoluminescence spectra (c) and time-resolved photoluminescence spectra (d) of the prepared samples.

The separation and migration of photogenerated carriers and the formation of active oxygen species are also very important in the photocatalytic oxidation of TL. Nyquist impedance plots (Supplementary Fig. 25a) and photocurrent response curves (Supplementary Fig. 25b) show that the prepared 1.8 Ni/BWO sample has the smallest resistance and the highest photocurrent density. Photoluminescence spectra (Supplementary Fig. 25c) and time-resolved photoluminescence spectra (Supplementary Fig. 25d) also show the lower carrier recombination and longer carrier lifetime of 1.8 Ni/BWO. These results indicate that the separation and migration of photogenerated carriers is promoted by Ni doping and shows a volcano-type trend with increasing Ni dopant content. This is related to the surface O_V, according to the reported studies, which is considered as the electron enrichment center for the capture of photogenerated electrons.

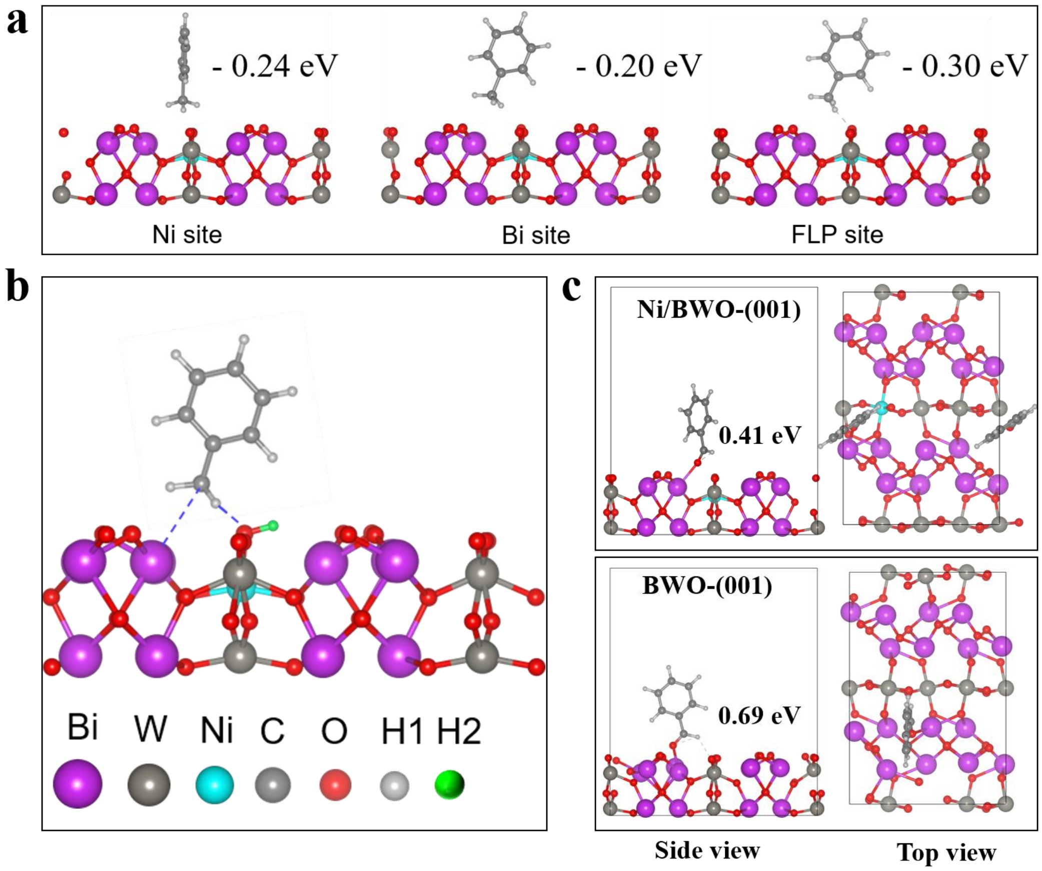

Supplementary Fig. 26 The specific optimized adsorption structures of the C-H bond in toluene on different surface sites (a). The stable configuration of C_7H_7 intermediate adsorbed on Ni/BWO-(001): Green H atom is from the deprotonation of toluene (b). The stable configuration of benzaldehyde adsorbed on Ni/BWO-(001) and BWO-(001) with the desorption energy (c): the bigger desorption energy indicates the more difficult desorption of benzaldehyde.

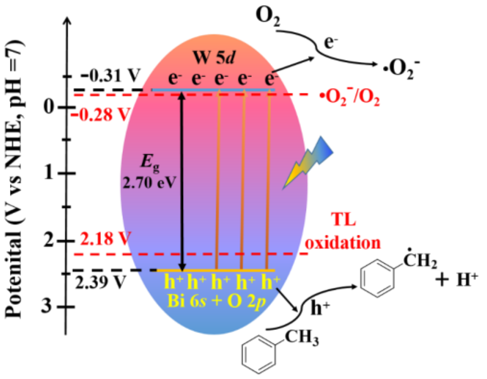

$C_6H_5CH_3 + h^+ \rightarrow C_6H_5CH_2\cdot + H^+$

$O_2 + e^- \rightarrow \cdot O_2^-$

$C_6H_5CH_2\cdot + \cdot O_2^- \rightarrow C_6H_5CH_2OO\cdot^-$

$C_6H_5CH_2OO\cdot^- + H^+ \rightarrow C_6H_5CH_2OOH$

$C_6H_5CH_2OOH \rightarrow C_6H_5CHO + H_2O$ (major)

$2\ C_6H_5CH_2OOH \rightarrow C_6H_5CHO + C_6H_5CH_2OH + O_2$ (minor)

$C_6H_5CHO + 1/2\ \cdot O_2^- \rightarrow C_6H_5COOH$ (major)

Supplementary Fig. 27 Possible mechanism for the oxidation of toluene based on semiconductor energy band theory and photo-induced active radical.

A possible mechanism based on traditional semiconductor energy band theory and photo-induced active radical is proposed to illustrate the photocatalytic mechanism of 1.8 Ni/BWO for the photocatalytic oxidation of toluene. Firstly, the surface unsaturated W atoms are responsible for adsorbing oxygen molecules while FLPs would coordinate with the C-H bond of toluene via Bi·· C and O···H coordination. It makes the activation of substrates and builds bridges for the transfer of photogenerated carriers from the photocatalysts to toluene and O_2. Under photoexcitation, the electrons of Bi 6s and O2p orbitals will be excited and jump to the empty 5d orbitals of W, generating photo-induced electrons and hole pairs. The photogenerated electrons accumulate in $[WO_4]^{2-}$ layer and photogenerated holes are retained in $[BiO]^+$ layer. Due to the suitable energy band structure of prepared BWO and 1.8 Ni/BWO, their CB and VB potentials have the ability to reduce O_2 to $\bullet O_2^-$ (-0.28 V vs. NHE) and oxidize toluene (2.18 V vs. NHE), respectively.[4] In other words, the photogenerated holes of Bi 6s and O2p orbitals will be transferred to C-H bonds through Bi·· C and O···H coordination, attacking toluene molecules to dehydrogenate into carbon center radicals. The photogenerated electrons in the 5d orbitals of W will reduce the O_2 adsorbed on unsaturated W to $\bullet O_2^-$ free radicals which is one of active O species for oxidizing activated toluene molecules. Finally, free $\bullet O_2^-$ species could oxidize the intermediate

products such as carbon center radicals ($*C_7H_7$), benzyl alcohol step by step. Due to the unique surface active structure of 1.8 Ni/BWO, the main product is benzaldehyde. As the accumulation of benzaldehyde, it can be further oxidized to benzoic acid by $\bullet O_2^-$ species.[6, 14] This is consistent with our experiment results of toluene oxidation.

Supplementary Tables

Supplementary Table 1 The actual mass fraction of Ni calculated by ICP-MS results.

Sample	0.9 Ni/BWO	1.8 Ni/BWO	2.6 Ni/BWO	Ni/BWO-surface
Ni mass fraction (100%)	0.91	1.77	2.62	1.78

Supplementary Table 2 The fraction of W^{5+} and W^{6+} in the prepared samples.

sample	peak	Assignment	Fraction (%)
BWO	W 4*f*	**W^{6+}**	99
		**W^{5+}**	1
0.9 Ni/BWO	W 4*f*	**W^{6+}**	90
		**W^{5+}**	10
1.8 Ni/BWO	W 4*f*	**W^{6+}**	85
		**W^{5+}**	15
2.6 Ni/BWO	W 4*f*	**W^{6+}**	88
		**W^{5+}**	12

Supplementary Table 3 The fraction of O species in the prepared samples.

sample	peak	Assignment	Fraction
BWO	O 1*s*	O_L	95
		O_V	5
		O_{OH}	-
0.9 Ni/BWO	O 1*s*	O_L	89
		O_V	10
		O_{OH}	1
1.8 Ni/BWO	O 1*s*	O_L	83
		O_V	3
		O_{OH}	14
2.6 Ni/BWO	O 1*s*	O_L	86
		O_V	4
		O_{OH}	10

Supplementary Table 4 The fitting results of W L_3-edge XAFS spectra.

sample	path	CN	Distance(Å)	Ó²(Å²)
BWO	W-O	6	1.79±0.03	0.002
1.8Ni/BWO	W-O	5.1±1.5	1.79±0.03	0.001

Supplementary Table 5 Apparent quantum efficiency (AQE) of the prepared samples for toluene oxidation.

entry	catalyst	AOE (%)
1	BWO	1.34
2	0.9 Ni/BWO	3.06
3	1.8 Ni/BWO	6.01
4	2.6 Ni/BWO	3.35
5	BWO-bulk	0.26
6	BWO-Ov	1.74
7	Ni/BWO-Surface	1.29
8	BiOCl	0.12

Supplementary Table 6 photocatalytic oxidation of toluene derivatives.

Reactant	Product	Con. (%)	Sel. (%)
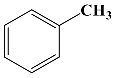	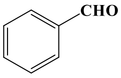	48	99
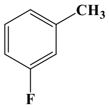	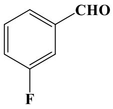	36	62
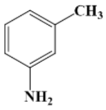	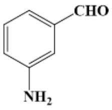	56	51
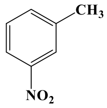	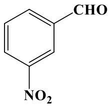	37	63
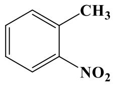	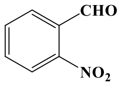	42	68
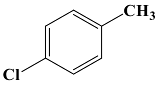	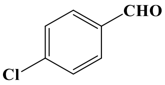	29	71

Reaction condition: 10 mg 1.8 Ni/BWO, 0.1 mmol toluene derivatives, 1.5 mL acetonitrile, O_2, reaction time 5h.

The experiments for the photocatalytic oxidation of toluene derivatives are conducted. Table S6 show that the toluene derivatives with electron-donating group (-NH_2) are easier to convert to the product than those with electron-withdrawing groups (-F, -Cl, -NO_2). The selectivity of the corresponding benzaldehyde derivatives is reduced due to the removal of other substitutions. The result shows that 1.8 Ni/BWO exhibits good conversion of other toluene derivatives.

Supplementary Table 7 The previous studies for the photocatalytic TL oxidation.

Catalyst	Reaction condition	Rate (μmol g^{-1} h^{-1})	Ref.
1.8 Ni/BWO	λ ≥ 400 nm, sample (10 mg), TL (2 mL),	4560	This work
$Bi_2W_{0.3}Mo_{0.7}O_6$	λ ≥ 420 nm, sample (15 mg), TL (1 mL)	1663	5
p-BWO	λ ≥ 400 nm, sample (250 mg), TL (8 mmol)	3453	6
p-BWO	λ ≥ 400 nm, sample (250 mg), TL (10 mmol),	4388	6
Fe(0.26)-BWO	λ ≥ 400 nm, sample (20 mg), TL (10 mmol)	1304	7
Pd/Bi_2WO_6	λ ≥ 400 nm, sample (50 mg), TL (1 mL)	1267	8
Flower-like Bi_2WO_6	λ ≥ 400 nm, sample (50 mg), TL (8 mmol)	464	9
HMNRs	No cut-off filter, sample (20 mg), TL (3 mL)	2254	10
$Cs_3Sb_2Br_9$	λ ≥ 420 nm, sample (10 mg), TL (5 mL)	2520	11
$Cs_3Bi_2Br_9$(1.7)/d-BiOBr	λ ≥ 400 nm, sample (10 mg), TL (5 mL)	7240	12
10wt%$Cs_3Bi_2Br_9$ /SBA-15	λ ≥ 420 nm, sample (10 mg), TL (5 mL)	12600	13
Fe-UiO-66	λ ≥ 380 nm, sample (10 mg), TL (5 μL)	1295	14
$0.01BiOCl/TiO_2$	λ = 365 nm, sample (25 mg), TL (1 mmol)	2000	15
Y_1/TiO_2	sample (50 mg), TL (0.5 mmol)	850	16
$Cs_4ZnSb_2C_{11}$	λ ≥ 400 nm, sample (10 mg), TL (1 mL)	1893	17
$Cs_2AgBiBr_6$/CN	sample (100 mg), TL (5 mL)	2630	18

We also compare the performance with the previously reported studies (Supplementary Table 7). It can be observed that 1.8Ni/BWO shows better performance than most other photocatalysts, indicating that creating CAUs via

in situ lattice substitution of Ni dopant is a high-efficiency strategy to improve the photocatalytic oxidation of TL. Moreover, the reported $Cs_3Bi_2Br_9$(1.7)/d-BiOBr and 10 wt% $Cs_3Bi_2Br_9$/SBA-15 exhibit much higher performance, suggesting that constructing heterojunction composites is also a feasible way to improve the photocatalytic efficiencies. However, introducing additional semiconductor materials would make the surface of photocatalysts become complex, causing the study of the surface reaction mechanism to be difficult.

Supplementary Table 8 The adsorption energy for O_2 on unsaturated Bi, Ni and W sites.

Site	Adsorption energy of O_2 (eV)
Bi	0.31
Ni	0.14
W	-0.10

Supplementary Table 9 The comparison of vibration frequency and corresponding force constant of the C–H bonds in TL molecules chemisorbed on the samples. The force constants (k) are calculated by molecular vibration equation $\upsilon = 1303(k/M)^{1/2}$. υ is vibration frequency from FTIR spectrum. M represents convert mass of C atom and H atom.

Sample	$\upsilon_{C\text{-}H}$ (cm^{-1})	k (N cm^{-1})	Sample	$\upsilon_{C\text{-}H}$ (cm^{-1})	k (N cm^{-1})	Sample	$\upsilon_{C\text{-}H}$ (cm^{-1})	k (N cm^{-1})
Ni/BWO	2944	5.54	BWO	2956	5.58	KBr	2958	5.59
	2920	5.44		2923	5.46		2927	5.47
	2875	5.28		2854	5.21		2854	5.21

The weakening degrees of the C–H bonds are quantized via the force constant (k). As shown in Table S9, after the chemisorption of TL molecules on

1.8 Ni/BWO, the values of k ($υ_{C-H}$) are 5.54, 5.44 and 5.28 N•cm^{-1}, respectively. Compared with the values of k ($υ_{C-H}$) on KBr, two C–H bonds are obviously weakened while one C-H bond is strengthened,[42] indicating that the products on FLPs may be easily converted to BD. This is consistent with the result of photocatalytic activity results. The changes in k ($υ_{C-H}$) values on BWO are small due to the absence of FLPs. The interaction between FLPs and TL would provide bridges for the migration of photogenerated holes from the catalyst to the –CH_3 group of toluene and the activated C–H bonds are easily broken and dehydrogenated.

Supplementary Table 10 The Bader charge of spatially separated Bi and O atoms in Ni/BWO-(001) and Ni-OH/BWO-(001) structure modes.

**Ni/BWO-(001)**		**Ni-OH/BWO-(001)**	
**Bi**	1.883	**Bi**	1.985
**O**	-0.438	**O**	-0.378

Supplementary Reference

1. Bai, Z. J. et al. $Cs_3Bi_2Br_9$ Nanodots Stabilized on Defective BiOBr Nanosheets by Interfacial Chemical Bonding: Modulated Charge Transfer for Photocatalytic C(sp^3)-H Bond Activation. *ACS Catal.* **12**, 15157–15167 (2022).

2. Lan, Z. A. et al. A Fully Coplanar Donor–Acceptor Polymeric Semiconductor with Promoted Charge Separation Kinetics for Photochemistry. *Angew. Chem. Int. Ed.* **60**, 16355–16359 (2021).

3. Nikoloudakis, E. et al. Dye-Sensitized Photoelectrosynthesis Cells for Benzyl Alcohol Oxidation Using a Zinc Porphyrin Sensitizer and TEMPO Catalyst. *ACS Catal.* **11**, 12075–12086 (2021).

4. Cao, S., Shen, B., Tong, T., Fu, J. & Yu, J. 2D/2D Heterojunction of Ultrathin MXene/Bi_2WO_6 Nanosheets for Improved Photocatalytic CO_2 Reduction. *Adv. Funct. Mater.* **28**, 1–11 (2018).

5. Zhang, K. et al. Two-dimensional $Bi_2W_xMo_{1-x}O_6$ solid solution nanosheets for enhanced photocatalytic toluene oxidation to benzaldehyde. *Appl. Catal. B Environ.* **315**, 121545 (2022).

6. Cao, X. et al. A photochromic composite with enhanced carrier separation for the photocatalytic activation of benzylic C–H bonds in toluene. *Nat. Catal.* **1**, 704–710 (2018).

7. Deng, X. X. et al. Boosted Activity for Toluene Selective Photooxidation over Fe-Doped Bi_2WO_6. *Ind. Eng. Chem. Res.* **59**, 13528–13538 (2020).

8. Yuan, B. et al. Photocatalytic aerobic oxidation of toluene and its derivatives to aldehydes on Pd/Bi_2WO_6. *Chinese J. Catal.* **38**, 440–446 (2017).

9. Liu, Y. et al. A green and efficient photocatalytic route for the highly-selective oxidation of saturated alpha-carbon C-H bonds in aromatic alkanes over flower-like Bi_2WO_6. *Chem. Commun.* **52**, 1274–1277 (2016).

10. Yu, B., Zhang, S. & Wang, X. Helical Microporous Nanorods Assembled by Polyoxometalate Clusters for the Photocatalytic Oxidation of Toluene. *Angew. Chem. Int. Ed.* **60**, 17404–17409 (2021).

11. Zhang, Z. et al. Revealing the A-Site Effect of Lead-Free $A_3Sb_2Br_9$ Perovskite in Photocatalytic C(sp^3)−H Bond Activation. *Angew. Chem. Int. Ed.* **59**, 18136–18139 (2020).

12. Ren, Y. et al. Photocatalytic synthesis of N-benzyleneamine from benzylamine on ultrathin BiOCl nanosheets under visible light. *J. Catal.* **380**, 123–131 (2019).

13. Dai, Y., Poidevin, C., Ochoa-Hernández, C., Auer, A. A. & Tüysüz, H. A Supported Bismuth Halide Perovskite Photocatalyst for Selective Aliphatic and Aromatic C–H Bond Activation. *Angew. Chem. Int. Ed.* **59**, 5788–5796 (2020).

14. Xu, C. et al. Turning on Visible-Light Photocatalytic C-H Oxidation over Metal-Organic Frameworks by Introducing Metal-to-Cluster Charge Transfer. *J. Am. Chem. Soc.* **141**, 19110–19117 (2019).

15. Wang, H. et al. Achieving High Selectivity in Photocatalytic Oxidation of Toluene on Amorphous BiOCl Nanosheets Coupled with TiO_2. *J. Am. Chem. Soc.* **145**, 16852–16861 (2023).

16. Xue, Z. et al. Efficient Benzylic C-H Bond Activation over Single-Atom Yttrium Supported on TiO_2 via Facilitated Molecular Oxygen and Surface Lattice Oxygen Activation. *ACS Catal.* **14**, 249–261 (2024).

17. Mai, H. et al. Synthesis of Layered Lead-Free Perovskite Nanocrystals with Precise Size and Shape Control and Their Photocatalytic Activity. *J. Am. Chem. Soc.* **145**, 17337–17350 (2023).

18. Song, J. et al. In situ growth of lead-free perovskite $Cs_2AgBiBr_6$ on a flexible ultrathin carbon nitride sheet for highly efficient photocatalytic benzylic $C(sp^3)-H$ bond activation. *Chem. Eng. J.* **453**, 139748 (2023).
